# Reducing Malaria Mortality at the Lowest Budget: An Optimization Tool for Selecting Malaria Preventative Interventions Applied to Ghana

**DOI:** 10.1177/2381468319861346

**Published:** 2019-07-25

**Authors:** Christophe Sauboin, Ilse Van Vlaenderen, Laure-Anne Van Bellinghen, Baudouin Standaert

**Affiliations:** GSK, Wavre, Belgium; CHESS In Health, Bonheiden, Belgium; CHESS In Health, Bonheiden, Belgium; GSK, Wavre, Belgium

**Keywords:** malaria interventions, RTS, S vaccine, Ghana, economics, optimization

## Abstract

*Background*. Preventative malaria interventions include long-lasting insecticidal nets (LLINs), indoor residual spraying (IRS), and seasonal malaria chemoprevention (SMC). The RTS,S vaccine candidate is now also approved for pilot introduction. This analysis estimates the optimal approach when combining current interventions with the vaccine to reduce under-five malaria mortality in Ghana at the lowest cost. *Methods*. A vector model was combined with a static human cohort model, using country-specific unit costs. Current coverage of each intervention was used as baseline. The base-case vaccine price was US$5/dose, with US$2 or US$10 tested in sensitivity analysis. Model simulations used a goal for extra mortality reduction in children aged <5 years, and identified the optimal combination of interventions to reach that goal at the lowest cost. The time horizon was 5 years. *Results*. The optimal sequence of investments would be the following: (1) introduce RTS,S; (2) introduce SMC; (3) increase LLINs and IRS concurrently. RTS,S introduction was associated with mortality reduction of 16% for a budget increase of US$15.6 million. Adding SMC with a partial coverage of 4% further reduced mortality by 1% at an additional budget of US$1.4 million. Subsequently scaling-up IRS, LLINs, and SMC at their maximum achievable coverage further reduced mortality by 82% (total reduction 98%) at an additional budget of US$474 million. At an RTS,S price of US$10/dose, SMC was first in the optimal sequence. A lower RTS,S price maintained the sequence but reduced the budget need. *Conclusions*. In Ghana, RTS,S introduction in addition to the existing measures would be the optimal first step for reducing under-five malaria mortality at the lowest cost, followed by SMC in relevant areas, and then further scaling-up of IRS and LLINs.

Malaria case incidence has fallen globally since 2010 according to World Health Organization (WHO) estimates, but the rate of decline has stalled since 2014.^1^ Malaria remains an important public health burden, particularly in sub-Saharan Africa, which accounts for about 90% of malaria cases and deaths worldwide.^1^ The number of malaria deaths in the WHO African region was estimated at 407,000 in 2016.^1^

Important progress has been made in terms of under-five mortality reduction over the last decades on the African continent. In Ghana, all-cause under-five mortality per 1,000 live births decreased from 155 (confidence interval [CI] = 139–171) in 1988 to 60 (CI = 52–68) in 2014 according to Demographic and Health Survey (DHS) surveys. The 2016 WHO Malaria Report showed estimates for the year 2015 of 7.3 million (range 4.8 million to 10 million) malaria cases and 13,000 (range 4,600 to 17,000) malaria-related deaths in Ghana.^2^ These estimates show the progress done regarding malaria mortality in the country with almost 50% reduction in comparison with the estimate of 25,000 malaria-related deaths reported for 2006 in the 2008 World Malaria report.^3^

Several interventions to prevent malaria are well established, including vector control methods such as insecticide-treated nets (long-lasting insecticidal nets [LLINs]), indoor residual spraying (IRS), and seasonal malaria chemoprevention (SMC).^2^ In Ghana, surveys reported LLIN usage of 52.2% in children and 9.7% of households covered with IRS (Table 2). Impact studies in other countries have shown the role of improved vector control and disease management for reducing malaria-related mortality.^4,5^

A Phase 3 study has been conducted with a malaria vaccine candidate showing efficacy against clinical malaria in two distinct age-groups receiving the first dose either at 6 weeks of age or between 5 and 17 months of age, with a higher vaccine efficacy demonstrated in the latter age-group. Its scientific name is RTS,S, which refers to its composition. RTS,S has received a positive scientific opinion from the European Medicines Agency and is now approved for pilot introduction. The WHO has recommended a series of evaluations in 3 to 5 distinct epidemiological settings in sub-Saharan Africa at sub-national level, covering moderate to high transmission settings.^6^ The recommended vaccine schedule for pilot introduction is based on the 5- to 17-month schedule with higher efficacy and consists of three primary doses at least 4 weeks apart, with the first dose given as close as possible to age of 5 months and the third dose completed by age of 9 months, followed by a fourth dose given 15 to 18 months after the third dose.^6^ In the present analysis, we have assumed a similar schedule and corresponding efficacy than the pilot introduction.

If recommendations are made for broader vaccine introduction, decision makers will need to decide which malaria interventions to prioritize to increase the health status of the population. Selecting the best investment strategy requires identifying an optimal mix of interventions to implement in order to achieve a specified public health goal at the lowest cost, depending on interventions already implemented and the characteristics of interventions in a given malaria setting. Fundamental questions from decision makers would be the following: 1) Should further investments in malaria interventions focus first on increasing the coverage of existing interventions or introducing new ones? 2) How should the mix of interventions be further expanded to reach a specific public health objective at the lowest budget?

Published cost-effectiveness studies have been conducted in Ghana regarding SMC,^7^ LLINs,^8^ and in the accompanying article on RTS,S. These analyses provide valuable information for decision makers; however, they don’t provide an explicit guidance on these questions because they don’t consider practical implementation constraints and budget aspects nor potential interactions between interventions to reach a defined goal.

A key objective is the reduction of malaria mortality in line with the Rollback Malaria Partnership objective to reduce malaria mortality by 90% by 2030. This will require information on the expected impact of different combinations of malaria preventative interventions and the associated costs in relation to available health care budgets.

The objective of the present study was to use a specific country as an example to illustrate how budget optimization modelling with country-specific input data can help estimate the optimal approach for combining preventative malaria interventions to reduce under-five malaria mortality. We selected Ghana as an example country, since it has areas of seasonal and nonseasonal malaria transmission and it already uses LLINs and IRS.

## Methods

### Model Overview

This analysis is based on a framework of constrained optimization as described in ISPOR good practices reports.^9,10^ This framework allows to assess combinations of interventions implemented simultaneously and take account of budget constraint. This budget optimization analysis used a static model consisting of two components, a vector model and a human host cohort model, each of which has been published separately.^11,12^

Using the current mortality estimate as baseline, the model simulations were run by setting a goal for the percentage mortality reduction in children aged <5 years, and identifying the optimal combination of interventions that would reach that specific goal at the lowest cost. Starting with an initial goal of 1% reduction, this process was repeated for a series of incremental mortality reductions in steps of 1 percentage point versus the baseline. At each step, the coverage for each intervention was set as a minimum constraint for the coverage at the next step, so that no intervention would be reduced in coverage. This approach guaranteed some level of continuity in pursuing an existing policy, avoiding drastic changes and policy reversals (e.g., reducing coverage of one intervention in order to invest released resources into expanding another intervention). Furthermore, it might not be possible to reduce or entirely remove an intervention for programmatic reasons. The time horizon of the analysis was 5 years, following a birth cohort up to 5 years of age. The model results were presented as graphs with the coverage of each intervention showing the optimal mix to achieve different targets in malaria mortality reduction at the lowest budgetary investments. The vector model was developed in MS Excel, and Matlab was used to develop the human host cohort model and the optimization algorithm.

In the following subsections, we describe 1) the vector model, 2) the human host cohort model, 3) the input data, 4) the optimization algorithm, and 5) the sensitivity analysis.

### Vector Model

The vector component was based on a previously published mathematical model that simulated mosquitoes’ behavior and mortality to estimate the impact of LLINs and IRS on human host availability and hazards to mosquitoes.^11,12^ The protection provided by LLINs and/or IRS was expressed as a change in the entomological inoculation rate (EIR), the number of infectious mosquito bites per person-year.^11^ Table 1 shows the key input parameters used in the vector model.

**Table 1:** Key Input Parameters Used in the Vector Model

Parameter	Value	Source
EIR in absence of intervention	71	Calibration result
Vector diversion from unprotected host	0.1	11
Vector mortality on attacking unprotected host	0.1	11
Daily vector survival probability when resting after feeding, unprotected by IRS	0.9	11
Efficacy of protection by LLIN		
Proportion of exposure during which the net is in use	0.9	11
Excess diversion from protected human by LLIN	0.44	12
Excess mortality upon attacking LLIN-protected human	0.46	12
Efficacy of protection by IRS		
Excess diversion from a protected human by IRS	0.56	12
Excess mortality upon attacking an IRS-protected human	0	
Relative risk of daily survival while resting after attacking an IRS-protected human	0.76	12

EIR, entomological inoculation rate; IRS, indoor residual spraying; LLIN, long-lasting insecticidal net.

Coverages for LLIN and/or IRS use were increased from 0% to 100% in increments of 10 percentage points, thereby producing 1,111 different coverage combinations. The vector model calculated the expected reduction in EIR resulting from each of these coverage combinations. This EIR was then used as an input into the human host cohort model.

### Human Host Cohort Model

Using the vector model’s estimated EIR associated with each of the 1,111 LLINs and IRS coverage combinations as input data, the human host model simulated the effect of vaccination and SMC on malaria mortality. It thus estimated the combined effect on malaria mortality of all four interventions. The human host model was the same static Markov cohort model as used in the cost-effectiveness analysis described in a companion paper, adapted to connect with the vector model and to take account of seasonal malaria and SMC. The connection consisted in converting the EIR resulting from the vector model into a corresponding force of infection (factor *q*) used as an input in the cohort model, which was calibrated to generate the corresponding incidence for uncomplicated clinical malaria, severe malaria, and mortality at varying transmission intensities as detailed in a previous publication.^13^ The reduction in malaria mortality resulted from a reduction in the force of infection in protected individuals.

For seasonal malaria areas, seasonality was modeled by multiplying the force of infection in the cohort model by a seasonality factor, obtained from the following equation:

(1) $$\mathrm{Seasonality}\;\mathrm{factor}\;=\;2*(1-(\mathrm{cosine}(\pi *{\mathrm{time}))}^{2}$$

SMC was only included in the model for the population living in two regions in North Ghana (Upper West and Upper East). These regions experience seasonal malaria, based on Malaria Atlas Project (MAP) population data in subnational administrative regions (Admin-1 data)^14^ and seasonality as defined by the WHO. Together, these regions account for 6.8% of the population of Ghana. The effect of SMC was calibrated based on a systematic review of data from over 12,000 participants in seven clinical trials.^15^ In the cohort model, the effect of SMC or vaccination is represented as a reduction in the force of infection. This differs from, and is usually greater than, the efficacy measured in clinical trials, which usually describes the reduction in symptomatic malaria episodes over a follow-up period. The effect of SMC reported in the systematic review^15^ was equivalent to an infection risk reduction of 90% in the model resulting in an average effectiveness of 73% across low, moderate, and high transmissions. However, the trials included in the systematic review were conducted in areas with highly seasonal malaria transmission, while Ghana has lower seasonality. In the northern regions of Ghana, the malaria transmission season lasts 6 to 7 months, with 50% to 60% of cases concentrated in the period from July to November.^7^ SMC is normally administered for 3 to 4 months,^7^ and thus its effectiveness will be lower in Ghana than in a more highly seasonal area because of the longer transmission season in Ghana. With the same infection risk reduction, the resulting SMC effectiveness was about 66% instead of 73%.

The four-dose vaccination schedule was assumed to be given at 6, 7.5, 9, and 27 months of age, focusing on child vaccination schedule, which showed to have a higher clinical efficacy than the infant schedule starting at 6 weeks of age. The effect of the RTS,S vaccine on the risk of infection was modeled as described in the companion paper on cost-effectiveness analysis for that specific schedule. A significant impact of RTS,S on mortality was not observed in the phase III trial in which access to treatment was optimized. However the potential impact on mortality was indirectly inferred from the cohort model based on 1) vaccine efficacy against clinical malaria cases and 2) the reported incidence of severe cases in the control arm of the trial and published case-fatality rate. It should be noted that the case-fatality rate for current analysis was scaled down to reproduce the estimated number of malaria-related deaths in Ghana reported in the WHO malaria report 2016.

### Input Data

Current coverage of malaria interventions was taken from StatCompiler^16^ for Ghana, based on DHS data (Table 2).

**Table 2:** Coverage Data for Malaria Interventions

	DHS 2014	MIS 2016
Children <5 years who slept under LLIN		52.2%
Households with IRS in the past 12 months	9.7%	
Households with at least one LLIN for every two persons and/or IRS in the past 12 months	50.4%	
Existing LLINs used last night	48.6%	
Children with fever who took artemisinin-based combination treatment	48.5%	

DHS, Demographic and Health Surveys; IRS, indoor residual spraying; LLIN, long-lasting insecticidal net; MIS, Malaria Indicator Survey.

The costs per person covered for LLINs, IRS, SMC, and vaccination were derived from published literature. All costs were expressed in 2015 US dollars. Where necessary, US dollar prices were converted to Ghanaian Cedi at the exchange rate of their calendar year, corrected for inflation based on the Consumer Price Index of Ghana and converted to US dollars using the 2015 exchange rate between Ghana Cedi and US dollar (US$1 for CEDI 3.668 in 2015 based on World Bank data).^17^

Evaluation of an LLIN distribution campaign in Ghana estimated the total cost of the campaign at US$23,848,034 (2012 US$), and the additional number of people sleeping under an LLIN at 2,216,980.^8^ Assuming that each LLIN lasts 3 years, this implies an annual cost of US$3.59 per person per year, including the cost of nets that are not used. Dividing the additional number of people sleeping under an LLIN by the number of LLINs delivered (3,664,028) indicates that 60.5% of LLINs were used; however, as some of the new LLINs may have replaced older LLINs, usage may have been higher. Procurement prices for LLINs appear to have reduced by 33% since 2012, based on data from UNICEF, and taking the price reduction into account resulted in an annual cost of US$2.23 per covered child, or US$11.13 for 5 years.

The annual cost of IRS was taken from Winskill et al.^18^: US$5.41 per person protected. This cost is applied to the whole covered population, and not only children. IRS can potentially protect all residents in a treated house, whereas the other interventions (LLINs, SMC, and RTS,S vaccination) are specifically targeted to children.

The cost for SMC was taken from a cost-effectiveness study in Ghana.^7^ The annual cost per SMC-covered child aged <5 years was US$9.66 (95% CI = 7.46–14.21) for four rounds of SMC, which equates to US$48.3 for 5 years.

For the RTS,S vaccination program, the cost per fully vaccinated child including vaccine cost and implementation cost was US$26.02, at a vaccine price of US$5 per dose, based on a study conducted in five African countries including Ghana.^19^

### Optimization Algorithm

The budget optimization analysis was conducted according to the following algorithm.

1. *Model calibration:* For simulating the effect of increasing coverages of LLIN and IRS, the vector model applies an estimated EIR in absence of any mosquito repellant/killing intervention. For the latter, an EIR value of 71 infectious bites per person-year provided the best match with the current number of malaria cases reported in the country when applying the closest approximates of the current coverages of interventions (54% LLINs, 10% IRS, and 48.5% access to artemisinin-based combination treatment [ACT]). This resulted in an estimate of 8.5 million cases of malaria at country level. This value is within the range reported in the 2016 World Malaria Report (data from 2015), which was 7.3 million (range 4.8 million to 10 million).^2^ The number of malaria deaths predicted by the model was 13,240, which matched the reported estimate of 13,000 (range 4,600–17,000).^2^ Given the higher variability in mortality, the calibration of the starting EIR in the model was based on the number of malaria cases rather than the number of malaria deaths. A coverage of 50% for LLINs was applied in the population aged >5 years, approximating 2014 DHS survey data (Table 2).
2. *Define optimization constraints:* Constraints on upper and lower coverage limits in the population aged <5 years were set for all interventions. The lower coverage limit was approximated at values of 10% for IRS and 54% for LLIN usage, based on a reported value of 52.2% for “children aged <5 years who slept under any net” (Table 2)^16^; this represents the current situation to which new interventions can be added and/or in which a scaling-up of LLIN and/or IRS can be performed. IRS, SMC, and RTS,S had an upper maximum achievable coverage of 90%, based on 2015 coverage in Ghana of 88% for the third dose of the diphtheria-tetanus-pertussis vaccine (DTP3).^20^ This differs from the cost-effectiveness analysis in the companion paper, where malaria vaccine coverage in children was set at Measles dose 1 coverage. Regarding LLINs, a survey conducted after a distribution campaign showed usage of 60.5% in persons having received a net. This usage rate was applied as a maximum achievable coverage in the model.
3. *Vector model optimum:* In the vector model, several different combinations of IRS and LLIN coverages can produce the same reduction in EIR, but have different budgets required to achieve these coverages. For all combinations of IRS and LLIN that respect the previously defined lower and upper coverage constraints and result in the same (reduced) EIR, only the combination with the lowest budget requirement was retained for that EIR. In total, 64 different values of EIR from 0.05 to 32 were used in the model. Malaria mortality was calculated for each EIR with the optimal combination of vector interventions (IRS and LLINs).
4. *Strategies for target mortality reduction:* For a given target mortality reduction, different types of strategies are tested: 1) keep LLINs/IRS combination only, 2) adding RTS,S to LLINs/IRS, 3) adding SMC to LLINs/IRS, and 4) adding a combination of RTS,S and SMC to LLINs/IRS. For all feasible strategies that allow reaching the target mortality reduction, the corresponding budget is calculated. Then the strategy with the lowest budget is selected.

The selection process described in point 4 is repeated with increasing targets of mortality reduction by increments of one percentage point, expressed as a percentage of baseline malaria mortality, that is, mortality in children below 5 years of age at current coverages of IRS and LLIN. When an optimal solution is found, the coverage constraints are adjusted for the next iteration in order to maintain each intervention coverage at least at the same level.

### Sensitivity Analysis: Alternative Scenarios

Scenario analyses tested the impact of vaccine prices of US$2 per dose and US$10 per dose. These equate to overall vaccine and implementation costs of US$11.1 and US$51.69 per fully vaccinated child, respectively, based on the same study used to estimate the cost per fully vaccinated child at the base-case vaccine price of US$5 per dose.^19^

Another scenario analysis tested the impact of restricting the analysis to seasonal areas where SMC is applicable, which might result in a different optimal sequence of investments.

## Results

### Base-Case Analysis

**Figure 1:**
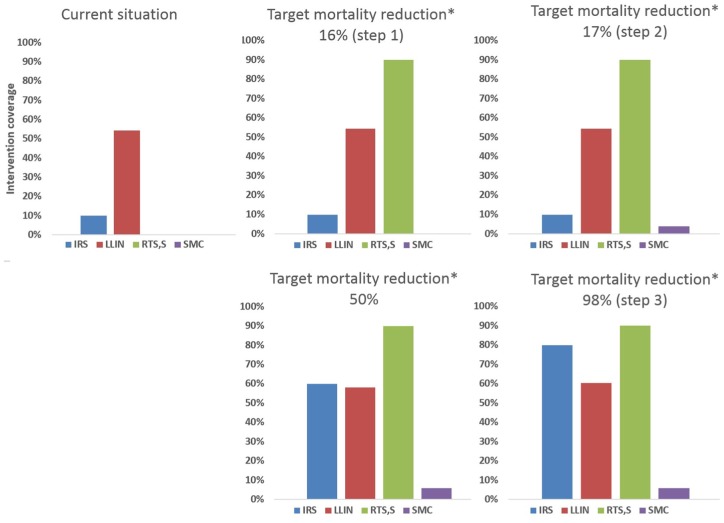
Evolution of coverage of malaria interventions with increasing targets for reduction of malaria mortality in children aged <5 years in Ghana. Base-case analysis (RTS,S vaccine price = US$5 per dose). IRS, indoor residual spraying; LLIN, long-lasting insecticidal net; SMC, seasonal malaria chemoprevention. (*) Target in comparison with malaria mortality in children aged <5 years in current situation. Current situation includes two interventions: IRS and LLIN (top-left graph). While these interventions are maintained, new interventions such as RTS,S and SMC are introduced to further reduce mortality by 16% (Step 1) then 17% (Step 2). For reaching higher mortality reduction targets (Step 3), further increases of SMC, IRS, and LLIN coverage would be required.

The malaria burden in children aged <5 years in Ghana estimated by the model with current levels of malaria interventions (54% LLIN, 10% IRS, and 48.5% with access to ACT) at an associated budget of US$83.5 million, was 8.5 million malaria cases and 13,240 malaria deaths. Figure 1 presents the optimal coverage for each intervention estimated by the optimization model at various levels of target mortality reduction in the base-case (RTS,S vaccine price = US$5 per dose). The optimal solution is shown for specific targets in mortality reduction in comparison with current situation: 16%, 17%, 50%, and 98%. Ninety-eight percent corresponds to the maximum achievable reduction in malaria mortality with the four preventive measures implemented at their maximum achievable coverage. For each target level, the optimal mix of interventions with their respective coverage is presented, that is, the mix achieving the target at the lowest budgetary investment. The optimal solution shows that further investment should be directed at introducing RTS,S vaccination (Step 1), in addition to maintaining the current coverages of LLIN and IRS. When RTS,S reaches its maximum achievable coverage of 90%, under-five mortality is reduced with approximately 16% compared to the current situation. Further target reductions in mortality resulted in increased SMC coverage but only to an intermediary level of about 60% of the children in seasonal areas (Step 2), its maximum achievable coverage being 90%. Because SMC is confined only to these geographic areas of Ghana with seasonal transmission, this resulted in only a small (approximately 1%) further reduction in mortality for all Ghanaian children aged <5 years, to about a 17% reduction from current mortality. Thereafter, further target reductions in mortality resulted in concurrent increases in both LLINs and IRS coverage above their current levels of implementation, together with bringing SMC to its maximum coverage in seasonal areas (Step 3). IRS implementation up to 80% coverage contributed the most to mortality reduction. IRS coverage started from a lower baseline coverage than LLINs (10% compared with 54%, respectively), and thus had a larger margin for increased impact. The extreme target of a maximum achievable mortality reduction of 98% required a combination of all interventions: 90% coverage with RTS,S, 80% coverage with IRS, 61% coverage with LLINs, and 85% coverage with SMC in the seasonal areas corresponding to 5.8% coverage at country level.

These results indicate that the optimal steps for reducing malaria mortality in children aged <5 years in Ghana would be first to introduce RTS,S vaccination up to maximum coverage in addition to maintaining the current coverage level of IRS and LLIN, then to introduce SMC in the seasonal transmission areas with a partial coverage of 60% in seasonal areas (below the maximum achievable coverage), and then to increase coverage of LLIN and IRS from existing levels, together with SMC. The cumulative mortality reduction and total budget associated with each step are shown in Table 3. The total budget includes the cost of preventive interventions and management costs for malaria-related outpatient and inpatient costs (health system perspective).

**Table 3:** Cumulative Mortality Reduction and Budget Increase for Optimal Sequence of Introduction of Malaria Interventions

Intervention Step	Cumulative Mortality Reduction From Current Mortality (%)	Cumulative Increase in Budget From Current Budget (US$)	Total Budget (US$)^a^
Current situation (reference level)	NA	NA	83.5 million
Step 1, introduce RTS,S vaccination up to maximum coverage (in addition to current situation)	16%	15.6 million	99.2 million
Step 2, add SMC in seasonal transmission areas up to intermediate coverage (in addition to completion of Step 1)	17%	15.6 million + 1.4 million	100.5 million
Step 3, concurrent increase in IRS and LLINs (in addition to completion of Step 2)	98%	15.6 million + 1.4 million + 473.6 million	574.1 million

IRS, indoor residual spraying; LLIN, long-lasting insecticidal net; NA, not applicable; SMC, seasonal malaria chemoprevention.

a Total budget includes the cost of preventive interventions and malaria management cost for outpatient visits and hospitalizations. Due to rounding, there might be a difference for the last digit between the budget increase and the total budget, the total budget corresponds to the correct rounding method.

**Figure 2:**
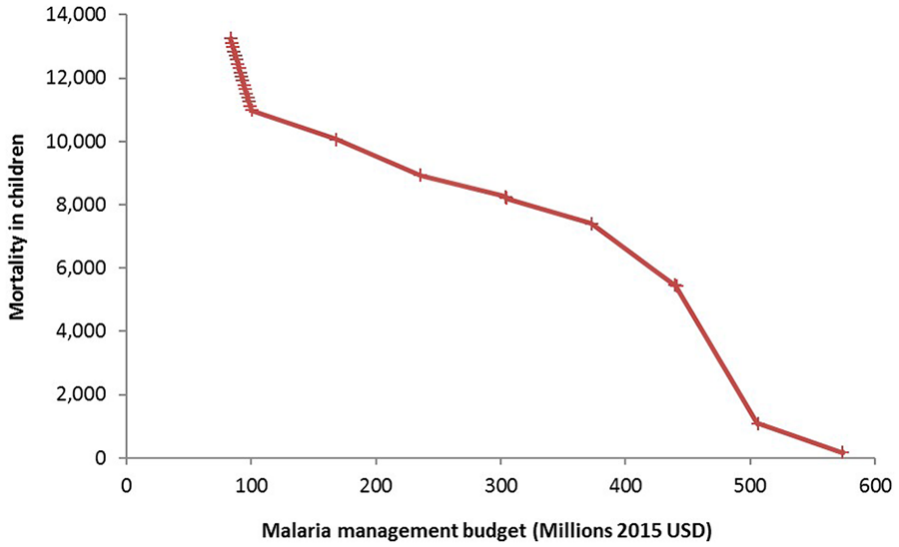
Evolution of malaria mortality in children aged <5 years in Ghana with increasing malaria management budget, according to the optimal sequence of intervention introduction derived from Figure 1. Base-case analysis (RTS,S vaccine price = US$5 per dose).

Figure 2 shows the estimated changes in malaria mortality in children aged <5 years with an increasing budget (including the costs of prevention and savings made on treatment costs). The initial steep fall in mortality from 13,240 cases in the current situation to approximately 11,120 cases represents the impact of Step 1 of the optimal intervention sequence above, that is, introduction of RTS,S vaccination up to its maximum coverage. The impact of Step 2, that is, introduction of SMC in the seasonal transmission areas but with partial coverage, is too small to see in the graph because it applies to only a small part of the population. The slower decline in mortality in the rest of the graph represents the impact of Step 3 of the optimal sequence, that is, increasing coverage of LLINs and IRS as well as SMC. The steps visible in the curve in this part of the graph reflect the increments of 10 percentage points by which coverage of LLINs and IRS is increased in the optimization model. It can be seen that Step 1 (vaccination introduction) was associated in the model with a steeper fall in mortality (i.e., a larger mortality reduction per unit of budget increase) than Step 3 (increase of LLIN, IRS, and SMC coverage).

### Scenario Analyses

Running a scenario analysis with a price of US$2 per dose for the RTS,S vaccine had no effect on the optimal sequence for introduction of each intervention estimated by the optimization model. The overall budget to reach the maximum mortality reduction would be reduced by about US$10.2 million compared with the base-case for which the total budget was estimated at US$574 million.

In a scenario analysis with the RTS,S vaccine price set at US$10 per dose, the optimal sequence for introducing interventions would change and SMC would be the first intervention in which to invest, followed by RTS,S vaccination. However, although SMC would be the first intervention in this scenario, its impact would remain small because it is confined only to the limited geographic area of Ghana with seasonal transmission. In this scenario, the overall budget increased by US$17.5 million in comparison with the base-case.

When restricted to seasonal areas where SMC can be applied, the optimal sequence of interventions remained unchanged. At a vaccine price of US$5 per dose, SMC remained the second intervention to be scaled-up after having reached the maximum achievable RTS,S coverage.

## Discussion

This budget optimization analysis using country-specific estimates for coverages and costs of malaria interventions indicates that, in order to reduce malaria mortality in children aged <5 years in Ghana at the lowest cost, the optimal sequence for introduction of malaria interventions in addition to the current coverage levels achieved with LLINs and IRS would be to introduce RTS,S vaccination first up to its maximum coverage, then to introduce SMC to intermediate coverage level in the areas with seasonal malaria transmission, and then to increase LLINs and IRS concurrently with SMC.

This sequence remained unchanged when the analysis was confined to the seasonal transmission areas of Ghana only. However, because SMC is relevant in only a limited geographic area of Ghana, representing 6.8% of the population, its contribution to the overall malaria mortality reduction in Ghanaian children aged <5 years would be limited to 2%.

Our model results indicate that RTS,S would be expected to have a larger impact on malaria mortality than increasing LLIN coverage. This finding may be explained by the limited remaining margin for increasing impact on transmission by scaling up LLIN coverage, as LLIN coverage is already around 50% in Ghana.

Our results differ from those reported in an evaluation of the relative cost-effectiveness of malaria interventions in sub-Saharan Africa conducted by Winskill et al.^18^ In this study, scaling up LLINs was the first in the sequence of interventions, with SMC second in seasonal settings, IRS second in nonseasonal settings with a parasite prevalence of 5% to 65%, and RTS,S vaccination second in nonseasonal settings with parasite prevalence of 65% or higher.^18^ The two analyses used different outcomes; our analysis focused on malaria mortality reduction in children aged <5 years in line with the Rollback Malaria Partnership objective to reduce malaria mortality by 90% by 2030, whereas the primary outcome measure considered by Winskill et al. was the number of malaria cases averted over a 10-year period in the entire population.^18^ Winskill et al. reported other outcome measures such as disability-adjusted life-years averted or the number of cases averted in children aged 6 months to 5 years, which also resulted in LLIN scale-up as the first intervention.^18^ Our analysis used costs specific to Ghana, and these differed substantially from the costs used by Winskill et al.,^18^ which could explain the differences in conclusions. Winskill et al. used a lower cost for LLINs of US$6.50 per child (compared with our cost estimate of US$11.13 per child over 5 years), a higher cost for RTS,S vaccination of US$39.25 per child (compared with our cost of US$26.02 per child), and a lower cost for SMC of US$4.95 per child per year (compared with our cost of US$9.66 per child per year).^18^ The cost for IRS was the same in both studies, at US$5.41 per person protected,^18^ but in our analysis this was applied to the whole covered population whereas the other interventions targeted only children aged <5 years. Winskill et al. found that their sequence of introduction and/or scale-up was sensitive to the price assumed for the RTS,S vaccine with an incremental cost-effectiveness ratio becoming comparable to IRS and SMC in their analysis as the vaccine price decreased.^18^ This may indicate that differences in the costs used in the two studies may help explain the different sequencing of interventions between Winskill et al. and our present analysis.

We may have underestimated the cost of IRS in our analysis. An evaluation of IRS in northern Ghana reported that the IRS program was reduced from 9 districts in 2012 to 4 districts in 2013 as a result of the switch from pyrethroid insecticides to more expensive organophosphates, although the national malaria-control program strategy still called for national scale-up of IRS.^21^ In our analysis, we took the cost of IRS from Winskill et al., which was based on use of dichlorodiphenyltrichloroethane (DDT) insecticides.^18^ Our results indicate that scale-up of IRS would be associated with a substantially larger budget increase compared with introduction of RTS,S vaccine. Furthermore, it is likely that this budget increase would be even higher if the cost for IRS were based on organophosphate insecticides. The potential effect of the cost of the insecticide used for IRS could be explored in future analyses.

Our model does not include transmission mechanisms such as those included in the model from Winskill et al. A reduction in the number of infected human hosts is expected to lead to a reduction of infected mosquitoes and in turn a reduction in the risk of infectious bites for humans. This would result in additional indirect protection for children when LLIN coverage is increased in adults. In our approach, only the coverage in children is varied. Another limitation is that we considered an overall national strategy for investments in malaria interventions whereas transmission intensity, access to care, and costs vary within the country. The only variation we accounted for is the seasonality in the northern regions of Ghana.

Other potential areas for future research include the effect of different insecticides and the impact of insecticide resistance on the potential effectiveness of LLINs and IRS. The vector model used in the present analysis was based on DDT parameters for the effect of IRS, with 56% mosquito diversion and 76% mosquito survival. Other insecticides, such as pyrethroids or organophosphates, may have different effects on mosquito behavior and mortality.

Finally, our analysis applied a uniform unit cost for each intervention regardless of coverage level. It is possible that unit costs could increase at higher levels of coverage, for example, if higher levels of coverage required roll-out to hard-to-reach populations. Winskill et al. found that including nonlinear functions to capture increasing costs at high coverage levels produced a more complex picture of the optimal intervention sequence, suggesting these nonlinearities are likely to have an impact on the optimal sequence of investments.^18^ Subtle changes in the inflection points could influence the mortality target at which a switch between interventions should be made, which may be critical to inform local planning.^18^

Our analysis uses country-specific coverage and cost input data to estimate the optimal sequence of introduction of malaria interventions in Ghana. By using inputs specific for other countries, or specific regions within a country, it could be used to produce customized estimates of the optimal sequence for a specific setting, allowing optimization of interventions according to local circumstances. The supplementary appendix summarizes the key questions and findings of the study and relevance for patient.

## Conclusions

This analysis used country-specific data for the coverage and cost of LLINs, IRS, SMC, and RTS,S vaccination in Ghana to estimate the optimal sequence of interventions to reduce malaria mortality in children aged <5 years. It found that the optimal sequence would be to introduce RTS,S vaccination first, followed by partially implementing SMC (below the maximum achievable coverage) in areas of seasonal transmission, followed by concurrent scaling up of SMC, LLINs, and IRS. The sequence remained the same when the analysis was restricted to the seasonal transmission areas of northern Ghana. RTS,S vaccination was associated with the steepest fall in malaria mortality per unit of budget increase, while scaling up of IRS would require a larger budget increase. The contribution of SMC to malaria mortality reduction in Ghana was small, because of its limited geographic relevance. The optimal sequence may vary between countries or between regions within a country, depending on specific local circumstances (e.g., intensity and seasonality of malaria transmission) and local costs. Our model provides a tool for developing customized estimates for specific countries and regions.

## Supplemental Material

Supplemental_Focus_on_Patient_REVISED.rjf_online_supp – Supplemental material for Reducing Malaria Mortality at the Lowest Budget: An Optimization Tool for Selecting Malaria Preventative Interventions Applied to Ghana Click here for additional data file. Supplemental material, Supplemental_Focus_on_Patient_REVISED.rjf_online_supp for Reducing Malaria Mortality at the Lowest Budget: An Optimization Tool for Selecting Malaria Preventative Interventions Applied to Ghana by Christophe Sauboin, Ilse Van Vlaenderen, Laure-Anne Van Bellinghen and Baudouin Standaert in MDM Policy & Practice

## References

[1] World Health Organization. World Malaria Report 2017 [cited 1 11, 2018]. Available from: http://who.int/malaria/publications/world-malaria-report-2017/report/en/

[2] World Health Organization. World Malaria Report 2016 [cited 4 10, 2017]. Available from: http://www.who.int/malaria/publications/world-malaria-report-2016/report/en/

[3] World Health Organization. World Malaria Report 2008 [cited 3 1, 2019]. Available from: https://www.who.int/malaria/publications/atoz/9789241563697/en/

[4] Eckert E Florey LS Tongren JE et al Impact evaluation of malaria control interventions on morbidity and all-cause child mortality in Rwanda, 2000–2010. Am J Trop Med Hyg. 2017;97(3 Suppl.):99–110. 28990918 10.4269/ajtmh.17-0281 PMC5619936

[5] Kayentao K Florey LS Mihigo J et al Impact evaluation of malaria control interventions on morbidity and all-cause child mortality in Mali, 2000–2012. Malar J. 2018;17(1):424. 30428880 10.1186/s12936-018-2573-1 PMC6236933

[6] World Health Organization. Malaria vaccine: WHO position paper—January 2016. Wkly Epidemiol Rec. 2016;91(4):33–51. 26829826

[7] Nonvignon J Aryeetey GC Issah S et al Cost-effectiveness of seasonal malaria chemoprevention in upper west region of Ghana. Malar J. 2016;15:367. 27423900 10.1186/s12936-016-1418-z PMC4947302

[8] Paintain LS Awini E Addei S et al Evaluation of a universal long-lasting insecticidal net (LLIN) distribution campaign in Ghana: cost effectiveness of distribution and hang-up activities. Malar J. 2014;13:71. 24581249 10.1186/1475-2875-13-71 PMC3944985

[9] Mauskopf J Standaert B Connolly MP et al Economic analysis of vaccination programs: an ISPOR Good Practices for Outcomes Research Task Force Report. Value Health. 2018;21(10):1133–49. 10.1016/j.jval.2018.08.005 30314613

[10] Crown W Buyukkaramikli N Thokala P et al Constrained optimization methods in health services research—an introduction: report 1 of the ISPOR Optimization Methods Emerging Good Practices Task Force. Value Health. 2017;20(3):310–9. 10.1016/j.jval.2017.01.013 28292475

[11] Killeen GF Smith TA Ferguson HM et al Preventing childhood malaria in Africa by protecting adults from mosquitoes with insecticide-treated nets. PLoS Med. 2007;4(7):e229. 17608562 10.1371/journal.pmed.0040229 PMC1904465

[12] Chitnis N Schapira A Smith T Steketee R Comparing the effectiveness of malaria vector-control interventions through a mathematical model. Am J Trop Med Hyg. 2010;83(2):230–40. 10.4269/ajtmh.2010.09-0179 PMC2911164 20682861

[13] Sauboin CJ Van Bellinghen LA Van De Velde N Van Vlaenderen I Potential public health impact of RTS,S malaria candidate vaccine in sub-Saharan Africa: a modelling study. Malar J. 2015;14:524. 26702637 10.1186/s12936-015-1046-z PMC4690265

[14] Malaria Atlas Project. *Plasmodium falciparum* parasite rate in 2–10 year olds 2015 Available from: http://www.map.ox.ac.uk/explorer/#/

[15] Meremikwu MM Donegan S Sinclair D Esu E Oringanje C Intermittent preventive treatment for malaria in children living in areas with seasonal transmission. Cochrane Database Syst Rev. 2012;(2):CD003756. 10.1002/14651858.CD003756.pub4 PMC6532713 22336792

[16] STATcompiler. Available from: http://statcompiler.com/en/

[17] World Bank. Official Exchange Rates. Washington: World Bank; 2016.

[18] Winskill P Walker PG Griffin JT Ghani AC Modelling the cost-effectiveness of introducing the RTS,S malaria vaccine relative to scaling up other malaria interventions in sub-Saharan Africa. BMJ Glob Health. 2017;2(1):e000090. 10.1136/bmjgh-2016-000090 PMC5321383 28588994

[19] Sicuri E Alonso S Fakih B et al Cost of implementation of malaria vaccination programmes in five sub-Saharan African countries (Burkina Faso, Kenya, Ghana, Mozambique and Tanzania). Paper presented at: AfHEA 4th Scientific Conference; October 10, 2016; Rabat, Morocco.

[20] World Health Organization. WHO/UNICEF estimates of national immunization coverage 2015 Available from: https://www.who.int/immunization/monitoring_surveillance/routine/coverage/en/index4.html

[21] Coleman S Dadzie SK Seyoum A et al A reduction in malaria transmission intensity in northern Ghana after 7 years of indoor residual spraying. Malar J. 2017;16(1):324. 28797269 10.1186/s12936-017-1971-0 PMC5553800

