# Supplementary figures and images for: Reducing Malaria Mortality at the Lowest Budget: An Optimization Tool for Selecting Malaria Preventative Interventions Applied to Ghana
Source: MDM Policy Pract. 2019 Jul 25;4(2):2381468319861346. doi: 10.1177/2381468319861346 (PMC6659186; doi:10.1177/2381468319861346)

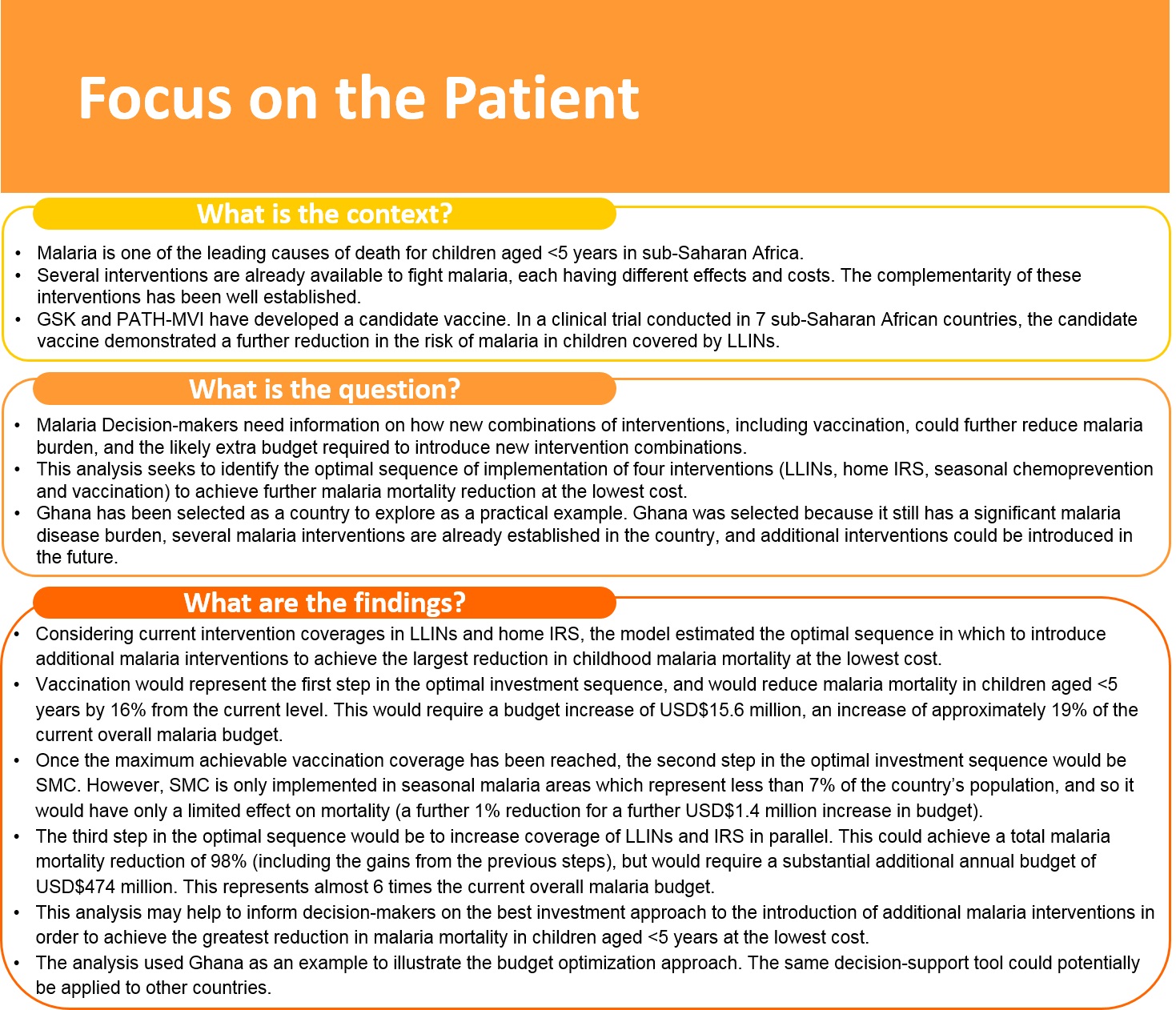

Supplement: Supplemental_Focus_on_Patient_REVISED.rjf_online_supp – Supplemental material for Reducing Malaria Mortality at the Lowest Budget: An Optimization Tool for Selecting Malaria Preventative Interventions Applied to Ghana [file Supplemental_Focus_on_Patient_REVISED.rjf_online_supp.jpg]
